# Supplementary material for: Chemical Modification of Cytochrome C for Acid-Responsive Intracellular Apoptotic Protein Delivery for Cancer Eradication
Source: Pharmaceutics. 2024 Jan 4;16(1):71. doi: 10.3390/pharmaceutics16010071 (PMC10819283; doi:10.3390/pharmaceutics16010071)
Supplement: Supplementary file 1 [file pharmaceutics-16-00071-s001.zip › pharmaceutics-2753718-supplementary.pdf]

Supporting Information

Chemical Modification of Cytochrome C for Acid-Responsive Intracellular Apoptotic Protein Delivery for Cancer Eradication

Bo Tang, Kwai Man Lau, Yunxin Zhu, Chihao Shao, Wai-Ting Wong, Larry M. C. Chow*, Clarence T. T. Wong*

Department of Applied Biology and Chemical Technology and State Key Laboratory of Chemical Biology and Drug Discovery, The Hong Kong Polytechnic University, Kowloon, Hong Kong, China.

Contents

Experimental Section

Figure S1. A) Reverse-phase HPLC chromatogram and B) MALDI-TOF/TOF mass spectrum of synthesized **CPP**.

Figure S2. A) Reverse-phase HPLC chromatogram and B) MALDI-TOF/TOF mass spectrum of synthesized **f-CPP**.

Figure S3. MTT cell viability assay of different reagents against HT29 cell line.

Materials and equipment

Rink Amide Resin was procured from CS Bio (Shanghai) Ltd. The *N*-α-Fmoc-protected amino acids were obtained from GL Biochem (Shanghai) Ltd. High-quality chemicals including *N,N*-dimethylformamide (DMF), dimethyl sulfoxide (DMSO), dichloromethane (DCM), diethyl ether, and acetonitrile (ACN) were purchased from Anaqua Global International Inc. Limited in ACS grade except ACN with HPLC grade. Piperidine was obtained from the Health and Safety Office of the Hong Kong Polytechnic University. Reagents such as 1-[bis(dimethylamino)methylene]-1H-1,2,3-triazolo-[4,5-b]pyridinium 3-oxide hexafluorophosphate (HATU), *N,N*-diisopropylethylamine (DIPEA), trifluoroacetic acid (TFA), and 2,3-dimethylmaleic anhydride were purchased from J&K Scientific Ltd. The Annexin V-FITC/PI Apoptosis Assay kit was obtained from Beijing Labgic Technology Co., Ltd. Thiazolyl Blue Tetrazolium Bromide (MTT) was purchased from Avantor® VWR International, LLC. The Promega CellTiter-Glo® Luminescent Cell Viability Assay Kit, Roswell Park Memorial Institute (RPMI) 1640 Medium, Fetal bovine serum (FBS), and Penicillin-Streptomycin were procured from Fisher Scientific (Hong Kong). Cytochrome C (equine) was purchased from Sigma Chemical Co. Phosphate-buffered saline (PBS), Hanks Balanced Salt Solution (HBSS), and Hoechst 33342 were acquired from Phygene Biotechnology Co., Ltd. The Human Colorectal Adenocarcinoma Cell Line (HT29) was obtained from the American Type Culture Collection (ATCC no. HTB-38). Nude Mice were purchased from the Hong Kong Polytechnic University Centralised Animal Facilities. All animal experiments were approved by the Animal Experimentation Ethics Committee of the University.

Electrospray ionization (ESI) mass spectra were recorded on an Agilent 6540 liquid chromatography -electrospray ionization quadrupole-time-of-flight mass spectrometer. Matrix-assisted laser-deprotection/ionization time-of-flight/time-of-flight (MALDI-TOF/TOF) mass spectra were recorded using a Bruker UltrafleXtreme MALDI-TOF/TOF mass spectrometer. The analytical reverse-phase HPLC separation was performed on a Waters XTerra™ MS C18 column (5 µm, 3.9 mm × 150 mm) at a flow rate of 0.4 mL min^{-1} using a Waters Acquity H-class system equipped with a photodiode array detector. The preparative reverse-phase HPLC separation was performed using a MACHEREY-NAGEL NUCLEODUR C18 HTec column (5 µm, 21 mm × 250 mm) at a flow rate of 7 mL min^{-1} using a Waters HPLC system equipped with a Waters 1525 binary pump and a Waters 2998 photodiode array detector. The solvents used for HPLC analysis were of HPLC grade. The condition used for the analysis was set as follows: solvent A = 0.1% TFA in ACN and solvent B = 0.1% TFA in deionized water; gradient: 5% A + 95% B in the first 5 min, and then changed to 100% A + 0% B in 25 min, maintained under this condition for 5 min, changed to 5% A + 95% B and maintained under this condition for 5 min.

Synthesis of cell-penetrating peptide Fmoc-GCKKLFKKILKKL-$CONH_2$ (CPP)

N-α-Fmoc-protected amino acids, HATU, DIPEA, and rink amide resins were first weighed and dissolved in the corresponding volume of DMF. 20% (v/v) piperidine in DMF was used to remove Fmoc protecting groups. All reagents were loaded into the CEM Liberty Blue Automated Microwave Peptide Synthesizer. The reaction cycle was performed by the machine in the default mode. After washing with DMF and DCM three times, the resins were treated with a solution containing 97% TFA, 3% triisopropylsilane (TIPS) for 2 h for removal of resin and protecting groups from the peptide. Then the solid resin was removed through filtration and the filtrate was

added into cold diethyl ether drop-wisely for peptide precipitation. The solid was collected through centrifugation at 4000 rpm for 10 min and redissolved in 20% (v/v) ACN in H_2O.crude peptide was purified by reverse-phase HPLC followed by lyophilization. For **CPP**: HRMS (MALDI-TOF/TOF): *m/z* calcd for $C_{89}H_{147}N_{20}O_{15}S_1$ $[M+H]^+$, 1768.107; found, 1765.755.

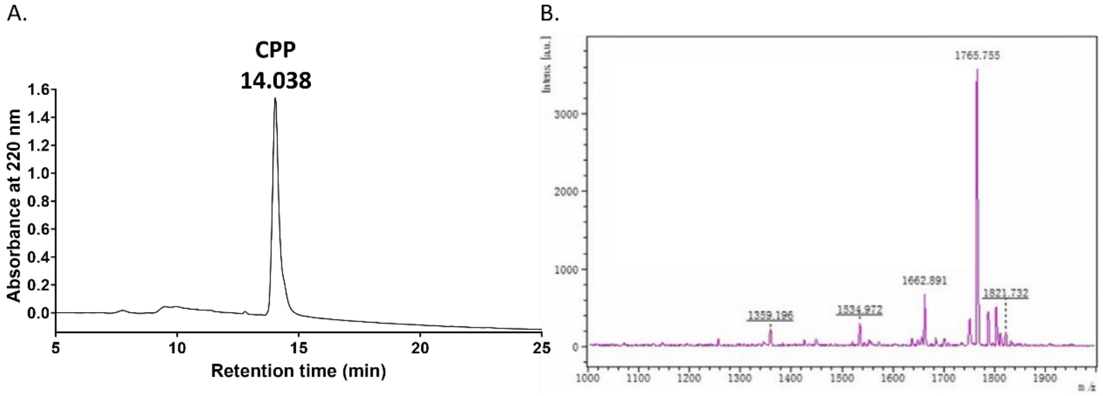

Figure S1. A) Reverse-phase HPLC chromatogram and B) MALDI-TOF/TOF mass spectrum of purified **CPP**.

Synthesis of fluorescein-tagged cell-penetrating peptide(f-CPP)

The peptide fluorescein-GCKKLFKKILKKL-$CONH_2$ (**f-CPP**) was synthesised by CEM Liberty Blue Automated Microwave Peptide Synthesizer by the method similar to that of **CPP** except the N-terminal Fmoc protecting group of **CPP** was deprotected by 20% (v/v) piperidine in DMF and an extra 5(6)-carboxyfluorescein was conjugated to the terminal amine of the peptide on resin by the peptide synthesiser. The deprotection and purification of **f-CPP** were performed as described in the synthesis of **CPP**. For **f-CPP**: HRMS (ESI): *m/z* calcd for $C_{95}H_{149}N_{20}O_{19}S$ $[M+3H]^{3+}$, 635.3671; found, 635.3678.

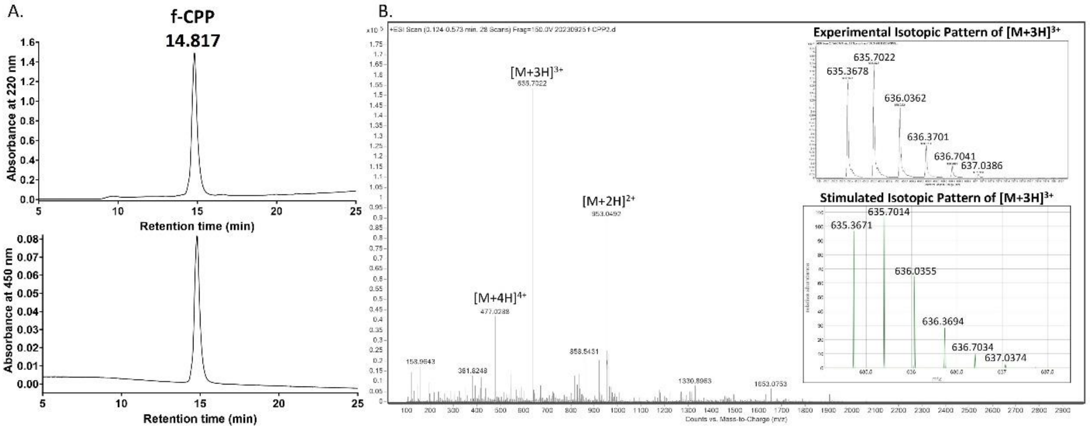

Figure S2. A) Reverse-phase HPLC chromatogram and B) MALDI-TOF mass spectrum of synthesised **f-CPP**.

Confocal microscopic analysis

A fluorescein-conjugated cell-penetrating peptide (**f-CPP**) was used to label cytochrome C for fluorescence microscopy-based visualisation of cellular internalisation kinetics. HT29 human colorectal adenocarcinoma cells cultured in RPMI 1640 medium supplemented with FBS (10%) and penicillin-streptomycin (100 unit mL^{-1} and 100 μg mL^{-1}, respectively), were seeded in 35 mm glass-bottom confocal dishes at a density of 1 × 10^5 cells per dish and incubated at 37 °C in a

humidified 5% CO_2 atmosphere for 24 hr to allow cell adhesion. The culture medium was then replaced with serum-free medium containing 20 μM fluorescent labelled **CPP-CytC** with or without DMA and incubated at 37 °C in a humidified 5% CO_2 atmosphere at pH 7.4 or 6.5 for 4 hr to permit endocytosis of the conjugate. Subsequently, the proteins-containing medium was removed, and cells were stained with 1 μg mL^{-1} Hoechst 33342 nuclear dye (Phygene) at 37 °C for 10 min. Cells were washed three times with PBS to remove excess dye. Localisation of intracellular cytochrome C was analysed using a Leica TCS SP8 MP Multiphoton/Confocal Microscope equipped with solid-state 488 nm and 405 nm lasers. Fluorescein on the protein was excited at 488 nm and its fluorescence emission was monitored at 500-530 nm. Hoechst 33342 was excited at 405 nm with emission detected at 415-480 nm. Digital images were captured and analysed using Leica Application Suite X image analysis software. This assay allowed the qualitative assessment of cytochrome C uptake dynamics in HT29 cells.

Cell viability assay

RPMI 1640 medium was adjusted to pH 7.4 and 6.5 by the addition of 2 M HCl. The medium was then sterile filtered through a 0.22 μm syringe filter. HT29 colon adenocarcinoma cells in RPMI 1640 medium supplemented with FBS (10%) and penicillin-streptomycin (100 unit mL^{-1} and 100 μg mL^{-1}, respectively) were seeded in a clear 96-well flat-bottom plate at a density of 2×10^4 cells per well and cultured at 37°C in a humidified 5% CO_2 atmosphere for 24 h to allow cell adhesion. The culture medium was replaced with a serum-free medium at pH 7.4 or 6.5 containing different concentrations of **CPP-CytC** with or without DMA ranging from 0 to 64 μM (0 μM, 1 μM, 2 μM, 4 μM, 8 μM, 16 μM, 32 μM,\ and 64 μM). Bovine serum albumin (BSA) was used as a control protein and modified by **CPP** and DMA following the same protocol for cytochrome C. The same concentration range of **CPP-BSA** with or without DMA in pH 7.4 or 6.5 was studied. Additional controls included untreated cells, 64 μM native cytochrome C, 0.64 mM **CPP** alone, and 64 μM cytochrome C with linker only. Following 24 h incubation, plates were centrifuged at 400 x g for 5 min. Supernatants were discarded, and cells were washed with PBS. A 100 μL volume of 0.5 mg mL^{-1} 3-(4,5-dimethylthiazol-2-yl)-2,5-diphenyltetrazolium bromide (MTT) reagent was added to each well and the plates were incubated for 4 h at 37 °C. The MTT solution was replaced with 50 μL DMSO for 10 min to solubilize the purple formazan crystals formed. Absorbance was read at 492 nm using Thermo Scientific Varioskan LUX Multimode Microplate Reader. Data were analyzed in GraphPad Prism 9 software. This assay assessed **f-CytC** cytotoxicity over a range of concentrations and pH conditions. Figure S3. showed the results of the MTT cell viability assay of 64 μM of cytochrome C or cytochrome C conjugates, or 0.64 mM of **CPP** against the HT29 cell line.

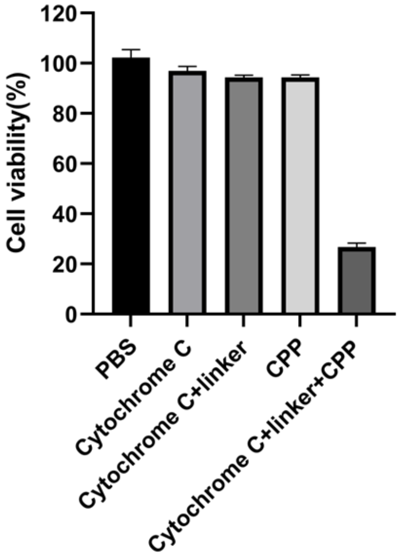

Figure S3. MTT cell viability assay of different reagents against HT29 cell line.

Table S1. The zeta potential of the CPP, cytochrome C and its analogs.

Sample	pH values	Zeta potential (mV)
CPP	7.4	14.2±6.74
	6.5	26.5±0.1
Cytochrome C	7.4	5.7±4.97
	6.5	4.65±1.12
CPP-CytC	7.4	16.7±7.62
	6.5	19.6±1.23
DMA.CPP-CytC	7.4	-1.16±5.09
	6.5	22.4±7.88
